# Diagnosis of Aspergillosis in Horses

**DOI:** 10.3390/jof9020161

**Published:** 2023-01-25

**Authors:** Radim Dobiáš, Petr Jahn, Katarina Tóthová, Olga Dobešová, Denisa Višňovská, Rutuja Patil, Anton Škríba, Pavla Jaworská, Miša Škorič, Libor Podojil, Michaela Kantorová, Jakub Mrázek, Eva Krejčí, David A. Stevens, Vladimír Havlíček

**Affiliations:** 1 Department of Bacteriology and Mycology, Public Health Institute in Ostrava, 702 00 Ostrava, Czech Republic; 2 Department of Biomedical Sciences, Faculty of Medicine, University of Ostrava, 703 00 Ostrava, Czech Republic; 3 Equine Clinic, University of Veterinary Sciences, 612 42 Brno, Czech Republic; 4 Department of Biology and Ecology, Faculty of Science, University of Ostrava, 710 00 Ostrava, Czech Republic; 5 Institute of Microbiology of the Czech Academy of Sciences, 142 20 Prague, Czech Republic; 6 Department of Analytical Chemistry, Faculty of Science, Palacký University, 771 46 Olomouc, Czech Republic; 7 Department of Pathology and Parasitology, University of Veterinary Sciences Brno, 612 42 Brno, Czech Republic; 8 Department of Molecular Biology, Public Health Institute in Ostrava, 702 00 Ostrava, Czech Republic; 9 California Institute for Medical Research, San Jose, CA 95128, USA; 10 Division of Infectious Diseases and Geographic Medicine, Stanford University School of Medicine, Stanford, CA 95128, USA

**Keywords:** equine aspergillosis, invasive pulmonary aspergillosis, bronchoalveolar lavage fluid, tracheal wash, serum, galactomannan, (1,3)-β-D-glucan, fungal secondary metabolites, equine herpes virus-5, equine asthma, guttural pouch

## Abstract

Invasive pulmonary aspergillosis (IPA) may be a rare cause of granulomatous pneumonia in horses. The mortality of IPA is almost 100%; direct diagnostic tools in horses are needed. Bronchoalveolar lavage fluid (BALF) and serum samples were collected from 18 horses, including individuals suffering from IPA (n = 1), equine asthma (EA, n = 12), and 5 healthy controls. Serum samples were collected from another 6 healthy controls. Samples of BALF (n = 18) were analyzed for *Aspergillus* spp. DNA, fungal galactomannan (GM), ferricrocin (Fc), triacetylfusarinin C (TafC), and gliotoxin (Gtx). Analysis of 24 serum samples for (1,3)-β-D-glucan (BDG) and GM was performed. Median serum BDG levels were 131 pg/mL in controls and 1142 pg/mL in IPA. Similar trends were observed in BALF samples for GM (Area under the Curve (AUC) = 0.941) and DNA (AUC = 0.941). The fungal secondary metabolite Gtx was detected in IPA BALF and lung tissue samples (86 ng/mL and 2.17 ng/mg, AUC = 1).

## 1. Introduction

Saprophytic filamentous fungi of the genus *Aspergillus* are common in the environment and can infect plants, insects, birds, and mammals [1,2]. *Aspergillus* spp. infections cause many diseases, from localized to fatal disseminated invasive infections in humans and animals [2,3]. In animals, aspergillosis is primarily a respiratory infection that may secondarily become systemic. Dominant species causing aspergillosis in horses are *A. fumigatus*, *A. niger*, *A. flavus*, *A. nidulans*, and *A. versicolor* [4].

Aspergilli commonly cause guttural pouch mycosis in horses (GPM). To the best of our knowledge, no comprehensive data on the prevalence of GPM has been published. During the last decade, GPM occurrences are mainly reported by the results of cluster case series [5,6,7,8]. The rare equine invasive pulmonary aspergillosis (IPA) has almost 100% mortality [9,10]. IPA risk factors include enteritis, colitis, prolonged administration of antibiotics, leukopenia, neutropenia, endocrinopathies, neoplasia, and salmonellosis (Supplementary Table S1). However, direct diagnostic tools for IPA in horses are needed.

Because equine IPA diagnosis is challenging, veterinarians should be aware of clinical and epidemiological settings in which the disease might develop [11]. Methods enabling reliable diagnosis of this disease using fungal biomarkers and economically sustainable therapeutic options could significantly improve prognosis. To this end, we here adopt a full suite of techniques for diagnosing human IPA for use in horses. Specifically, we combined culturing of fungi from bronchoalveolar lavage fluid (BALF) with the determination of *Aspergillus* DNA, galactomannan (GM), ferricrocin (Fc), triacetylfusarinin C (TafC) and gliotoxin (Gtx) in horses with IPA or with equine asthma (EA), and healthy horses, and compared the results of these analyses. GM and (1,3)-β-D-glucan (BDG) were determined in serum samples from horses of all three groups (IPA, EA, and control horses). The first aim of the study was to identify indicators distinguishing equine IPA, equine asthma (EA), and healthy horses. The second aim was the possibility of detecting *Aspergillus*-specific siderophores and fungal secondary metabolites during the diagnosis and therapy of GPM.

## 2. Materials and Methods

### 2.1. Study Design, Participants, Sample Processing

**Figure 1:**
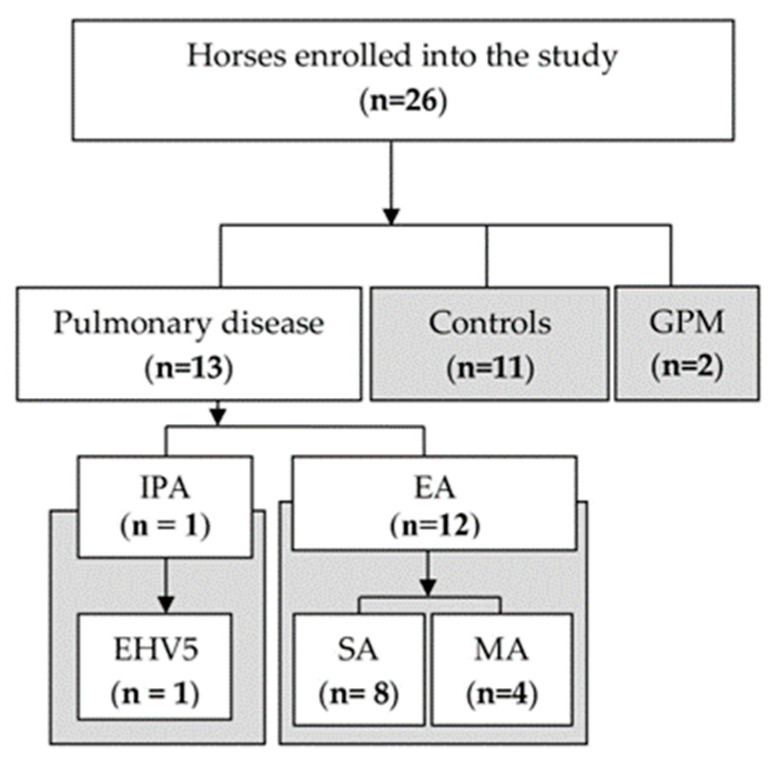
Flowchart of the study. IPA—Invasive Pulmonary Aspergillosis; GPM—Guttural Pouch Mycosis; EHV5—Equine Herpes Virus 5; EA—Equine Asthma; SA—Severe Asthma; MA—Moderate Asthma.

Clinical data, tracheal wash, BALF, samples collected from guttural pouches, and serum samples were collected prospectively from equine subjects stabled at an equine clinic (University of Veterinary Sciences Brno, Czechia) between February and June 2021. Twenty-six horses (Figure 1) were included in the study and divided into four groups: (1) IPA with Equine Herpes Virus 5 (EHV-5) pulmonary infection (n = 1); (2) equine asthma (EA) subdivided into severe (SA, n = 8) and moderate asthma (MA, n = 4); (3) healthy controls (n = 11) and (4) guttural pouch mycosis (GPM) (n = 2). 

#### 2.1.1. Horse with Invasive Equine Pulmonary Aspergillosis (IPA)

**Figure 2:**
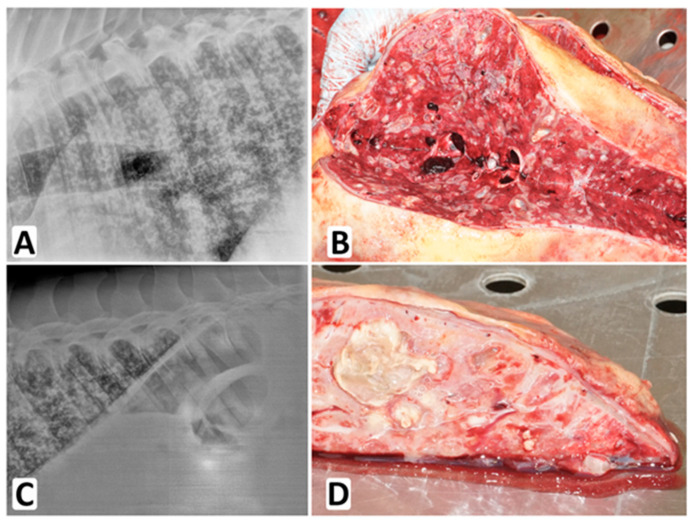
IPA in the equine lungs. Interstitial lung pattern and increased opacity of bronchial walls were recorded by X-ray imaging (**A**,**C**). Macroscopic *Aspergillus* lesions were found in the lungs during autopsy (**B**,**D**).

A three-year-old Slovak warmblood stallion was admitted to the equine clinic with acute colitis complicated later by equine multinodular pulmonary fibrosis (EMPF) and IPA. The diagnosis of EMPF was based on clinical signs (cough, persistent fever (38.5–41.0 °C), elevated respiratory rate (40–48/min), auscultation crackles and wheezes on the chest), lung ultrasound which revealed multiple comet tail artifacts in the lung field and consolidations in its dorsal parts, a lung radiograph which revealed an unstructured interstitial lung pattern (see Figure 2A,C) and detection of Equine herpes virus 5 in a tracheal wash by DNA analysis (PCR). IPA was suspected and serum for GM and BDG analysis was collected on day six, BALF for *Aspergillus* DNA, GM, Fc, TafC, and Gtx detection on day 14 before initiation of antifungal therapy. Five weeks after hospital admission, the stallion’s clinical condition began rapidly deteriorating and the horse was euthanized. The necropsy was performed and samples of lung, spleen, kidney, heart, pericardium, and mediastinal lymphonodes were examined histopathologically for the presence of *Aspergillus* DNA, GM, Fc, TafC, and Gtx.

#### 2.1.2. Horses with Equine Asthma (EA)

Twelve horses with EA were included in this group. All horses underwent physical examination and airway endoscopy with the transendoscopic collection of tracheal wash and bronchoalveolar lavage using a BAL tube. The enrollment into this study was based on EA symptoms, i.e., cough, tachypnea, or adventitious lung sounds lasting for at least two months, an endoscopic finding of secretions in the trachea, and cytology of BALF (more than 5% of cells in the BALF were neutrophils). This group was further divided into two subgroups. Eight horses (3 Warmbloods, 2 Quarterhorses, 1 Thoroughbred, 1 Shagya arab, and 1 Kladruber; age 9–20 years) were diagnosed with severe equine asthma based on signs of expiratory dyspnoea present during the examination or in the history and cytology of BALF (more than 25% of cells in the BALF were neutrophiles). Moderate asthma was diagnosed in 4 horses (2 Warmbloods, 1 Thoroughbred, and 1 Lippizaner; age 8–14 years) without expiratory dyspnoea, and BALF cytology (percentage of neutrophiles in BALF was 5–25%). Twelve tracheal wash and BALF samples of these horses were cultured, and the BALF samples were used for the analysis of Aspergillus-DNA, GM, Fc, TafC, and Gtx. Serum samples were examined for GM and BDG.

#### 2.1.3. Healthy Controls

Eleven horses (6 Warmbloods, 2 Thoroughbreds, 1 Pony, 1 Cold-blood, and 1 Cross-bred; age 2–22 years) without clinical signs of disease were used as healthy controls. Serum samples were collected from all of them and used to detect GM and BDG. Five horses of this group (4 Warmbloods, 1 Cross-bred; age 3–22 years) underwent the same examination as the EA horses. Enrollment into healthy controls was based on the absence of clinical signs of respiratory disease, negative finding on airway endoscopy, and <5% neutrophiles in BALF, which was used to detect Aspergillus-DNA, GM, Fc, TafC, and Gtx.

#### 2.1.4. Horses with GPM

A cross-bred gelding aged 3.5 years (GPM 1) and a 3.5-year-old Hanoverian gelding (GPM 2) were admitted to the equine clinic after exhibiting signs of pharyngeal (GPM 1) or oral (GPM 2) dysphagia, fever and cough (GPM 2) were included in this group. Complete clinical examination, including the lower airways and hematological, biochemical, and lung ultrasonography analyses yielded no remarkable findings in horse GPM 1 and worsened nutritional status (spinous processes, ribs, and tuber coxae easily discernible, prominent tailhead and accentuated withers, shoulders, and neck), possible masseter atrophy, and bruxism in horse GPM 2.

An endoscopic examination of the upper airway revealed the presence of mycosis within the medial compartment of the left guttural pouch in horse GPM 1 and atypically localized small symmetrical mycotic-appearing lesions in the median compartments of both guttural pouches in the horse GPM 2. A plaque sample was collected trans-endoscopically on the day of admission in both horses, and on day 7 (GPM 1) and 15 (GPM 2). Both samples were used for culture, microscopy, and analysis for *Aspergillus*-DNA, GM, Fc, TafC, and Gtx. Left-sided double ligation of the internal carotid and occipital artery on the cardiac side was performed under inhalation anesthesia in horse GPM1 and endoscopically guided laser fenestration in the dorsal pharyngeal recess with openings to both guttural pouches in horse GPM 2 as a treatment. Both horses recovered

#### 2.1.5. Sample Processing, Galactomannan and (1,3)-β-D-glucan Assay

Tracheal wash, BALF specimens, serum, and material collected from guttural pouches and necropsy were examined at the Public Health Institute in Ostrava (Czechia) and the Czech Academy of Sciences. Aspergillus spp. isolates were grown on Sabouraud dextrose agar (Merck, Darmstadt, Germany), malt extract agar (Oxoid, Basingstoke, UK), or Czapek yeast agar (Oxoid, Basingstoke, UK) at 26 and 37 °C. BDG (Fungitell^®^, Associates of Cape Cod, Falmouth, MA, USA) and GM (Aspergillus EIA, Bio-Rad Platelia™, Marnes-la-Coquette, France) were quantified following the manufacturer’s instructions for use with human samples. BDG concentration was determined in pg/mL, GM was set as a routinely widely used positivity index (PI). Cut-off values for horses have not been previously described.

### 2.2. DNA Extraction, PCR, and Sequencing

PCR identified Aspergillus DNA in the tracheal wash, guttural pouches, and BALF samples. Fungal DNA was isolated using the QIAamp DNA Mini Kit (Qiagen, Hilden, Germany) following the manufacturer’s tissue protocol. Aspergillus spp. detection was performed using the AsperGenius^®^Species Multiplex real-time PCR kit (PathoNostics, Maastricht, The Netherlands) according to the manufacturer’s instructions. Species identification was performed based on micro- and macro-morphological characteristics and confirmed by sequencing internal transcribed spacer rDNA (specifically, the ITS1-5.8S-ITS2 cluster) [12]. The PCR reaction volume was 25 µL, and the final reaction mixture contained 1xTB Green Premix Ex Taq II (Takara Biomedicals, Osaka, Japan), 10 pmol of each primer pair for ITS1 and ITS4 [13], and 2 µL of isolated DNA. The PCR amplification temperature profile consisted of an initial denaturation (30 s at 95 °C), 40 cycles of amplification (5 s at 95 °C and 30 s at 60 °C), and final melting analysis (60–95 °C; reading was performed at 0.1 °C intervals) for product verification. The sequencing of PCR products was performed using the BigDye^®^ Terminator v1.1 Cycle Sequencing Kit (Thermofisher, Applied Biosystems™, Waltham, MA, USA) and an ABI 3130 genetic analyzer according to the manufacturer’s instructions. The obtained DNA sequences were paired using SeqScape (Thermofisher, Applied Biosystems™) software and compared to available DNA sequences using the Basic Local Alignment Search Tool and the CBS-KNAW (www.cbs.knaw.nl, accessed on February–June 2021) database.

### 2.3. Calibration Standards and Fungal Metabolite Extraction

Standards of Fc, TafC, and ferrioxamine E (FoxE) ferriforms were obtained from EMC Microcollections GmbH (Tübingen, Germany). Gtx was purchased from APExBIO (Houston, TX, USA). A high-performance liquid chromatography (HPLC) peptide standard mixture and leucine enkephalin (Leu-Enk) were purchased from Sigma-Aldrich (Prague, Czech Republic). Metabolites were extracted from serum and BALF samples using our previously reported two-step liquid-liquid extraction protocol [14]. Briefly, 50 µL samples were spiked with FoxE and Leu-Enk internal standards to final concentrations of 50 and 25 ng/mL, respectively, then extracted with ethyl acetate (200 µL) and dried under reduced pressure. The residual aqueous phase was mixed with methanol (200 µL) and kept at −80 °C for 1 h before centrifugation (14,000× *g*, 4 °C, 10 min) to remove precipitated proteins. The supernatant was then transferred to a vial containing the evaporated ethyl acetate fraction and concentrated under reduced pressure.

The serum and BALF samples from the control horse group were used to prepare matrix-matched calibration curves for Fc, TafC, and Gtx based on solutions with final concentrations of 1, 5, 10, 50, 100, and 250 ng/mL. FoxE and Leu-Enk were added as internal standards, and samples were extracted using the procedure specified above. Limits of detection (LOD) and limits of quantitation (LOQ) were calculated from the standard deviation of the response (Sy) and the slope (S) using the following equations: LOD = 3.3 × (Sy/S) and LOQ = 10 × (Sy/S).

Gtx was extracted from lung tissue homogenates using the above-mentioned extraction protocol and quantified by an assay using internal authentic standards. Briefly, whole lungs were lyophilized and homogenized with a mortar and pestle. A 25 mg sample of the resulting homogenized powder was then dissolved in (400 µL) 15% Liquid Chromatography/Mass Spectrometry (LC/MS) grade acetonitrile (ACN). All samples were analyzed in triplicate, and results are reported as means ± standard deviation (SD). A system suitability test with an HPLC peptide standard mixture was used to verify the method’s robustness. Calibration curves are presented in the Supplementary Materials (Supplementary Table S2).

### 2.4. Liquid Chromatography and Mass Spectrometry Analysis

Pooled samples of serum and BALF or lung homogenates were re-suspended in 150 or 200 µL of 15% ACN, respectively, and injected onto an Acquity HSS T3 C18 analytical column (1.8 μm, 1.0 × 150 mm, Waters, Milford, MA, USA). Gradient elution was performed at a 50 µL/min flow rate using solvents A (1% ACN with 0.1% formic acid) and B (99% ACN with 0.1% formic acid) with the following program: 0 min (2% B); 2 min (2% B); 9 min (60% B); 11 min (99% B); 14 min (99% B); 14.5 min (2% B); 20 min (2% B). Siderophores and toxins were analyzed using a Dionex UltiMate 3000 HPLC system (Thermo Fisher Scientific, Waltham, MA, USA) coupled with a SolariX 12T FTICR (Bruker Daltonik, Bremen, Germany). Data were collected in electrospray positive ion mode with the continuous accumulation of selected ions windows adjusted to 200–700 and 500–1500 Da using a quadrupole filter. Qualitative and quantitative data analysis was conducted using the in-house CycloBranch version 2.0.19 software [15] and Data Analysis 5.0 (Bruker Daltonik, Bremen, Germany).

### 2.5. Statistical Analysis

Quantitative data analysis, including calculation of medians and means, generation of boxplots, and statistical tests, was conducted with the R 4.0.2 package [16] using standard libraries. Optimal cut-off values were estimated using the R library ‘cutpointr’ [17] to draw Receiver Operating Characteristic (ROC) curves and to determine specificity and sensitivity. False negative and false positive rates were calculated based on IPA and non-IPA cases comparison. Confidence intervals for specificity and sensitivity were calculated using the epiR library [18].

## 3. Results

### 3.1. Horse with Invasive Equine Pulmonary Aspergillosis (IPA)

In the BALF sample, which was collected on day 14, *Aspergillus* spp. was detected by PCR. The GM concentration in BALF (PI = 2.56) provided evidence of aspergillosis, as did PCR of BALF samples and the serum BDG concentration (1142 pg/mL). No siderophores were found in the BALF, and serum GM (IP = 0.409) was inconclusive (Table 1).

**Figure 3:**
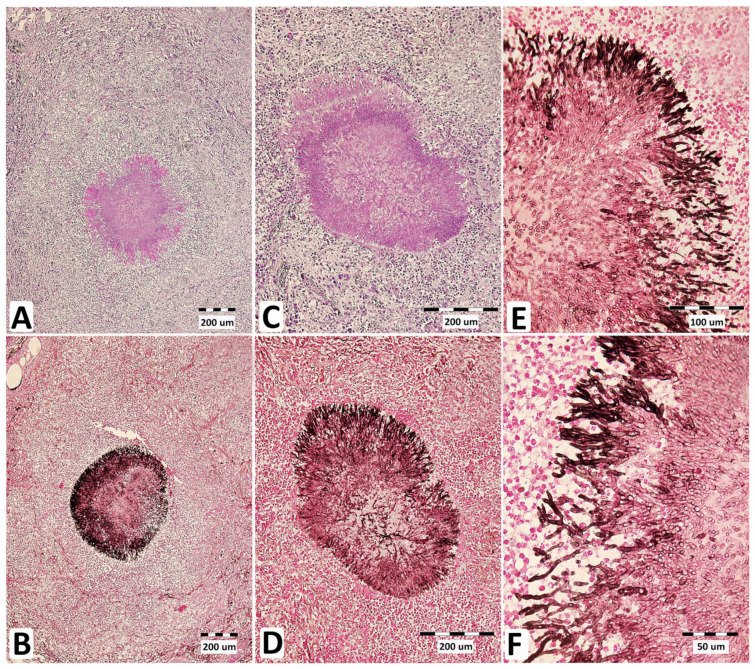
Micromorphology of *A. fumigatus* lung lesions in a stallion suffering from mycotic pneumonia. Central necrosis in pyogranuloma containing fungal elements visualized using periodic acid (Schiff stain) and Grocott’s methenamine silver (GMS) stain at 100× magnification (**A**,**B**) and 200× magnification (**C**,**D**), together with *Aspergillus* hyphae visualized by GMS staining at 600× magnification (**E**,**F**).

Macro- and microscopic histopathological examination of tissue samples confirmed the clinical diagnosis and mycotic pneumonia (Figure 2B,D, and Figure 3). Microscopic examination was positive for fungi only in the lungs. The kidney, heart, pericardium, and lymph nodes were unaffected. The brain was not examined.

**Figure 4:**
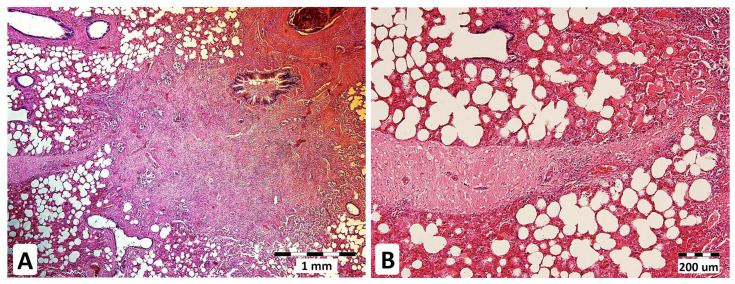
Chronic interstitial pneumonia in equine IPA. The formation of fibrous nodules in the lung parenchyma, HE stains, magnification 40× (**A**). Multifocal thickening of interalveolar septa with prominent fibrosis (indicated by a strand of fibrous tissue) in a lung parenchyma sample stained with hematoxylin-eosin (HE) (**B**) at 100× magnification.

*A. fumigatus* was found in the post-mortem specimens of lung, spleen, and liver tissues by culture, and fungal DNA was detected in the lungs and spleen. All the tests were negative except for Gtx in the BALF and lung tissue (86 ng/mL and 2.17 ng/mg). *A. fumigatus* superinfection on EMPF (Figure 4) initiated by EHV-5 infection was recorded as the primary cause of death (euthanasia).

### 3.2. Comparing Asthmatic and Healthy Equine Populations (non-IPA) to IPA

To evaluate the diagnostic utility of equine serology, we compiled a dataset based on serum and BALF samples from one horse with IPA (case 1), 12 horses with equine asthma (8 horses with SA, 4 horses with MA) and 11 healthy control horses (BALF only from 5 healthy control horses) representing the non-IPA cohort (Table 1). Only one tracheal wash of a healthy horse was colonized by *A. fumigatus* (Supplementary Table S3). In order of decreasing frequency, the *Aspergillus* species detected by culture were *A. niger*, *A. fumigatus, A. montevidensis*, *A. nidulans*, *A. flavus,* and *A. chevalieri. Aspergillus* spp. were not detected by culture in BALF of healthy controls.

**Figure 5:**
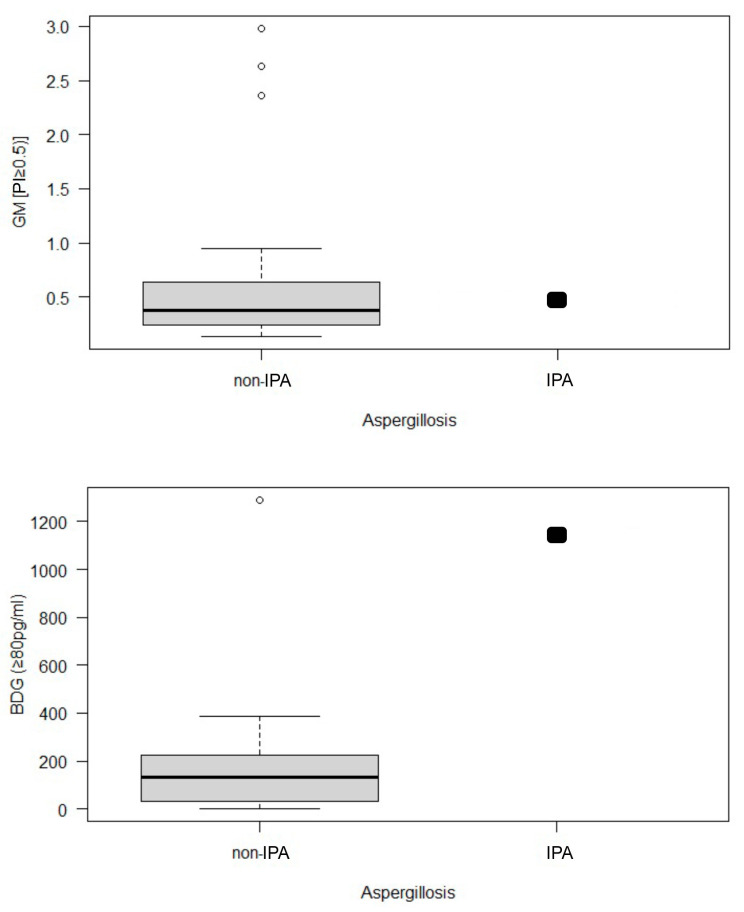
(Top) Serum biomarker concentrations in the group of horses without pulmonary aspergillosis (non-IPA, n = 23) and one horse with pulmonary aspergillosis. Serum galactomannan in the horse with pulmonary aspergillosis (index positivity = 0.478) and the control group (IQR = 0.375). (Bottom) serum (1,3)-β-D-glucan in the horse with invasive pulmonary aspergillosis (1142 pg/mL) and the healthy control group (131.5 pg/mL).

The median GM value in the BALF of the non-IPA group was 0.22, which is substantially lower than that recorded in the IPA case (PI = 2.56). However, a GM PI above 2.56 was obtained in a non-IPA horse with severe asthma resulting from *A. montevidensis* and *A. chevalieri* colonizing infection. This horse had BALF positive for *Aspergillus* spp. DNA but no evidence of colonization by *A. fumigatus*. Overall, GM serology showed inferior diagnostic performance: false-positive diagnoses were obtained for three subjects (14.3%), of which two had severe asthma, and one was healthy. The single IPA case had a positivity index similar to that of non-IPA horses (Figure 5), resulting in a low area under the curve (AUC) value (Table 2). Interquartile ranges (IQR) were also very close: two consecutive serum GM measurements (IQR = 0.478) in the IPA horse were similar to the median serum GM level in the non-IPA group (IQR = 0.375).

Conversely, serum BDG and BALF *Aspergillus*-DNA both distinguished between the IPA individual and the non-IPA group with high sensitivity, specificity, and NPV. The median serum BDG concentration for the non-IPA group was much lower (IQR = 131.5 pg/mL) than for the IPA case (IQR = 1142 pg/mL). The most significant outlier was a non-IPA horse with severe asthma and massive BALF colonization by non-*A. fumigatus* species (positive for *A. montevidensis* and *A. chevalieri* by PCR). Healthy controls would have been categorized as lacking a diagnosis of aspergillosis based on serum BDG if applying a cut-off of 131.5 pg/mL was used. *Aspergillus*-DNA offered lower specificities and positive predictive values because of the presence of *Aspergillus* spp. in the BALF of the healthy control horses without IPA.

### 3.3. Horses with GPM

In GPM 1 the GP tissue debridement sample analysis revealed the presence of *A. fumigatus* by culture, DNA, and microscopy. Intracellular fungal components, i.e., GM and Fc were present, but the extracellular fungal markers TafC and Gtx were not detected, indicating the presence of a non-proliferating localized fungal ball.

The sampling of the left guttural pouch repeated on day 7 revealed *A. nidulans* by culture with panfungal GM and Fc biomarkers (Table 3).

In GPM 2 a sample taken from a lesion during surgery revealed very high Fc and GM biomarker loads (Table 3) that were attributed to *A. nidulans* based on culture, *Aspergillus*-*DNA*, and microscopy data. Fifteen days after the surgery, a second sampling revealed reduced biomarker levels in both pouches. In the right guttural pouch, all diagnostic indicators (*Aspergillus*-DNA, Fc) were negative except GM, which remained elevated (Table 3), probably reflecting the cell wall contents of dead fungal cells.

## 4. Discussion

According to a review of the literature (Supplementary Table S1), the most common factor in equine invasive lung infection is a condition that weakens the animal’s immune system, e.g., severe enterocolitis or underlying lung pathology [9,19,20]. In EA, non-*fumigatus* species appear to be responsible for asthmatic sensitization.

The route of fungal infection of lung tissue in horses is not known, and two possibilities are considered. Some authors [19,21], suspect fungal spore inhalation to be the route of infection, and moldy hay and bedding are implicated as its source. However, a described association between enterocolitis and pulmonary aspergillosis suggests invasion of the damaged intestine as the route of systemic infection [22,23].

According to the Consensus Statement of the American College of Veterinary Internal Medicine [24], which was revised in 2016 [25], the BALF examination is the gold standard method for diagnosing Equine Asthma (Inflammatory Airway Disease, Recurrent Airway Obstruction). Our equine dataset showed that the trachea in horses could be massively colonized by *Aspergillus* species, especially in individuals with severe asthma (Supplementary Table S3). In such cases, *Aspergillus* spp. may be detected in BALF and tracheal samples.

Diagnosis of IPA is challenging in humans as well as in veterinary medicine. Revision and Update of the Consensus Definitions of Invasive Fungal Disease from the European Organization for Research and Treatment of Cancer and the Mycoses Study Group Education and Research Consortium highlight the importance of noninvasive diagnostic tests with the strong recommendation given to both serum and bronchoalveolar lavage fluid GM testing and detection of *Aspergillus* DNA by PCR [26]. Depending on the patient cohort, the sensitivity and specificity of GM testing in serum varies between 42–79%, and 85–86%, respectively [27,28]. In human IPA diagnosis, GM sensitivity and specificity for BALF samples range from 81–86%, and 88–91%, respectively [29,30,31].

GM detection for the diagnosis of systemic fungal infections in animals has been used with conflicting results. GM concentration in the serum of Magellanic penguins was shown to not discriminate healthy birds from those with pulmonary aspergillosis [32]. On the contrary, serum *Aspergillus* GM appeared as a noninvasive marker for the diagnosis of disseminated aspergillosis in dogs with a sensitivity of 92% and a specificity of 86% [33].

In our study, GM in BALF was a useful marker of *Aspergillus* invasion because the median GM level in the non-IPA control group differed significantly (*p* < 0.05) from that in the horse with IPA. Measurement of GM levels in BALF can provide close to 100% sensitivity and 94% specificity in the diagnosis of IPA, which is consistent with the results obtained using this approach in human intensive care (88% sensitivity and 87 % specificity) [26,29]. However, it should be noted that false positives may reduce the sensitivity and specificity values obtained for this technique in future studies examining larger populations [34]. This would make it necessary to use an integrated diagnostic strategy combining BALF GM measurements with other techniques. We, therefore, investigated the diagnostic utility of multiple non-culture methods that rely on fungal infection biomarkers used in human medicine [29,35,36,37]. The detection of these biomarkers in equine serum and urine samples warrants further study, and the available serum data are not conclusive other than in the case of serum BDG (Figure 5).

Serum BDG concentrations in most of the studied non-IPA horses (IQR = 131.5 pg/mL) were much lower than in the IPA individual (1142 pg/mL). One horse from the EA group had a strongly positive BDG concentration (1291 pg/mL). However, the horse in question had severe asthma with the colonization of the lower respiratory tract by *Aspergillus* species (*A. montevidensis* and *A. chevalieri*). Based on the performance of BDG as a diagnostic tool in human studies [35,38], this biomarker’s high NPV (92%) in horses suggests that it could be useful in diagnosing equine IPA. In contrast, the overall sensitivities achieved for serum-based IPA diagnosis using GM and BDG in a guinea pig model were just 68% and 46%, respectively, with specificities of 80% and 100%, respectively. In the guinea pig animal model study, PCR of blood samples provided the earliest indication of IPA, while increasing serum GM and BDG concentrations were indicators of disease progression. Accordingly, combining serum PCR with GM and BDG gave the best diagnostic performance regarding the area under the curve (AUC = 0.95) [39].

In our study, of the three fungal secondary metabolites used to diagnose invasive aspergillosis, only Gtx was detected in the BALF and lung tissue samples, confirming ongoing IPA. Gtx is an immunosuppressive fungal toxin that is secreted to the extracellular environment in response to fungal stress [40]. Studies have shown the utility of siderophores and/or gliotoxin in human blood and urine as useful biomarkers of *Aspergillus* infection, and we have detailed this recently [36,41,42,43].

In accordance with previous reports [4], *A. niger*, *A. flavus, A. nidulans,* and *A. versicolor* were the most common agents detected in EA. Moreover, *A. nidulans* was the dominant agent involved in both equine GPM cases examined in this work. Although combined occurrence with *A. fumigatus* was briefly observed in one GPM case, the dominance of *A. nidulans* was probably responsible for detecting the siderophore Fc in guttural pouch debridement samples.

Since IPA is relatively rare in horses, the dataset of our study is limited by the presence of only one horse with IPA. For firmer conclusions, further clinical studies will be needed; however, according to our results, the rising serum concentration of BDG in combination with the detection of DNA-*Aspergillus* spp. and GM in BALF could be useful in the early diagnosis of equine IPA. Before the fungal agent is identified by culture and for faster prediction of an active fungal infection in GP, early Fc detection in GP debridement could be helpful in combination with DNA-*Aspergillus* spp. test.

## Figures and Tables

**Table 1:** Horses enrolled in the study, IPA vs. control groups (n = 24).

Individual Equine Data	Sample	Culture	Microscopy (BSH)	PCR	GM (PI)	BDG (pg/mL)	Gtx (ng/mL)	Fc (ng/mL)	TafC (ng/mL)	Conditions
WB, S, 3 yrs.	BALF	*A. fumigatus*	Positive	*A. fumigatus*	2.56	NA	86	Negative	Negative	IPA, EHV-5, Colitis, Death
	Serum	NA	NA	NA	0.547	1142	Negative	Negative	Negative	
QH, G, 9 yrs.	BALF	Negative	Negative	Negative	0.1	NA	Negative	Negative	Negative	Severe EA
	Serum	NA	NA	NA	0.379	132	Negative	Negative	Negative	
QH, M, 13 yrs.	BALF	*A. fumigatus*, *A. montevidensis*, *A. flavus*, *A. niger*	Negative	Negative	2.12	NA	Negative	Negative	Negative	Severe EA
	Serum	NA	NA	NA	2.358	387	Negative	Negative	Negative	
TB, G, 9 yrs.	BALF	*A. montevidensis*	Negative	Negative	0.15	NA	Negative	Negative	Negative	Severe EA
	Serum	NA	NA	NA	0.175	0	Negative	Negative	Negative	
Sh, G, 13 yrs.	BALF	*A. nidulans*, *R. stolonifer*	Negative	Negative	0.10	NA	Negative	Negative	Negative	Severe EA
	Serum	NA	NA	NA	0.431	16	Negative	Negative	Negative	
WB, G, 14 yrs.	BALF	Negative	Negative	Negative	0.1	NA	Negative	Negative	Negative	Severe EA
	Serum	NA	NA	NA	0.16	0	Negative	Negative	Negative	
WB, M, 20 yrs.	BALF	Negative	Negative	Negative	0.1	NA	Negative	Negative	Negative	Severe EA
	Serum	NA	NA	NA	0.133	0	Negative	Negative	Negative	
KB, G, 13 yrs.	BALF	*A. montevidensis*, *A. chevalieri*, *A. alternata*	Negative	*Aspergillus* sp.	3.96	NA	Negative	Negative	Negative	Severe EA
	Serum	NA	NA	NA	2.983	1291	Negative	Negative	Negative	
WB, G, 13 yrs.	BALF	Negative	Negative	Negative	0.22	NA	Negative	Negative	Negative	Severe EA
	Serum	NA	NA	NA	0.943	204	Negative	Negative	Negative	
WB, G, 13 yrs.	BALF	Negative	Negative	Negative	1.3	NA	Negative	Negative	Negative	Moderate EA
	Serum	NA	NA	NA	0.155	103	Negative	Negative	Negative	
L, M, 1 yrs.	BALF	Negative	Negative	Negative	0.24	NA	Negative	Negative	Negative	Moderate EA
	Serum	NA	NA	NA	0.216	244	Negative	Negative	Negative	
TB, M, 14 yrs.	BALF	*A. montevidensis*, *A. fumigatus*, *A. alternata*	Negative	Negative	0.86	NA	Negative	Negative	Negative	Moderate EA
	Serum	NA	NA	NA	0.65	110	Negative	Negative	Negative	
WB, G, 8 yrs.	BALF	*A. alternata*	Negative	Negative	0.2	NA	Negative	Negative	Negative	Moderate EA
	Serum	NA	NA	NA	0.276	139	Negative	Negative	Negative	
CrB, G, 3 yrs.	BALF	Negative	Negative	Negative	0.31	NA	Negative	Negative	Negative	Healthy
	Serum	NA	NA	NA	0.342	248	Negative	Negative	Negative	
WB, M, 3 yrs	BALF	Negative	Negative	Negative	1.48	NA	Negative	Negative	Negative	Healthy
	Serum	NA	NA	NA	0.371	192	Negative	Negative	Negative	
WB, G, 22 yrs.	BALF	Negative	Negative	Negative	0.18	NA	Negative	Negative	Negative	Healthy
	Serum	NA	NA	NA	0.268	142	Negative	Negative	Negative	
WB, M, 13 yrs.	BALF	Negative	Negative	Negative	0.11	NA	Negative	Negative	Negative	Healthy
	Serum	NA	NA	NA	0.257	100	Negative	Negative	Negative	
WB, M, 9 yrs.	BALF	Negative	Negative	Negative	1.5	NA	Negative	Negative	Negative	Healthy
	Serum	NA	NA	NA	0.411	255	Negative	Negative	Negative	
WB, M, 11 yrs.	Serum	NA	NA	NA	0.621	9	Negative	Negative	Negative	Healthy
P, G, 2 yrs.	Serum	NA	NA	NA	2.629	43	Negative	Negative	Negative	Healthy
WB, G, 25 yrs.	Serum	NA	NA	NA	0.5	131	Negative	Negative	Negative	Healthy
CB, M, 21 yrs.	Serum	NA	NA	NA	0.606	397	Negative	Negative	Negative	Healthy
TB, M, 7 yrs.	Serum	NA	NA	NA	0.257	13	Negative	Negative	Negative	Healthy
TB, G, 6 yrs.	Serum	NA	NA	NA	0.105	88	Negative	Negative	Negative	Healthy

WB, warmblood; QH, quarter horse; Sh, Shagya; KB, Kladruber Black; L, Lipizzaner; CrB, Cross-bred; P, pony; CB, Cold-blood; S, stallion; G. gelding; M, mare; BSH, branching septated hyphae; GM, galactomannan; BDG, 1,3-β-D-glucan; Gtx, gliotoxin; Fc, ferricrocin; TafC, triacetylfusarinin C; IPA, invasive pulmonary aspergillosis; EHV-5, Equine Herpes Virus 5; EA, equine asthma; NA, not applicable.

**Table 2:** *Aspergillus* biomarkers in equine BALF and serum.

	bGM (PI = 2.56)	b*Aspergillus*-DNA	bGtx (86 ng/mL)	sGM (PI = 0.409)	sBDG (1142 pg/mL)
Sensitivity (95% CI)	100% (0.02–1)	100% (0.02–1)	100% (0.02–1)	100% (0.16–1)	100% (0.16–1)
Specificity (95% CI)	94% (0.71–1)	88% (0.64–0.99)	100% (0.8–1)	55% (0.32–0.77)	92% (0.62–1)
PPV	18% (0.01–0.99)	33% (0.01–0.91)	100% (0.02–1)	18% (0.02–0.52)	67% (0.09–0.99)
NPV	100% (0.79–1)	100% (0.78–1)	100% (0.8–1)	100% (0.72–1)	92% (0.72–1)
AUC	0.941	0.941	1	0.625	0.95

GM, galactomannan; b, bronchoalveolar lavage fluid; PPV, Positive Predictive Value; NPV, Negative Predictive Value; AUC, Area under the Curve; Gtx, gliotoxin; s, serum; BDG, 1,3-β-D-glucan; PI, Positivity Index.

**Table 3:** Diagnostic tools and *Aspergillus* biomarkers in equine guttural pouch debridement.

		Tool			Biomarker		
GP	Day	Culture	*Aspergillus*-DNA	Microscopy (BSH)	GM (PI ≥ 0.5)	Fc (ng/mL)	Sampling
GPM1 associated with nasopharyngeal dysphagia							
Left	1	*A. fumigatus*	*A. fumigatus*	Positive	9.72	492	BAT
Left	7	*A. nidulans*	*Aspergillus* spp.	Positive	10.4	1364	AS
GPM2 associated with oral dysphagia							
Left	1	*A. nidulans*	*Aspergillus* spp.	Positive	8.64	597	BAT
Right	1	*A. nidulans*	*Aspergillus* spp.	Positive	8.63	3574	BAT
Left	15	*A. nidulans*	Negative	Negative	7.54	496	AS
Right	15	Negative	Negative	Negative	7.65	ND	AS

GP, guttural pouch; GPM, guttural pouch mycosis; BSH, branching septated hyphae; GM, galactomannan; Fc, ferricrocin; BAT, before antifungal therapy; AS, after surgery; PI, positivity index; ND, not detected.

## Data Availability

Not applicable.

## Supplementary Materials

The following supporting information can be downloaded at: https://www.mdpi.com/article/10.3390/jof9020161/s1, Supplementary Table S1: Review of previously reported cases of invasive pulmonary aspergillosis in horses; Supplementary Table S2: Calibration curves for gliotoxin and ferricrocin; Supplementary Table S3: Characteristics of horses with underlying pulmonary disease, healthy controls, and horse with IPA.
