# Supplementary material for: Diagnosis of Aspergillosis in Horses
Source: J Fungi (Basel). 2023 Jan 25;9(2):161. doi: 10.3390/jof9020161 (PMC9966232; doi:10.3390/jof9020161)
Supplement: Supplementary file 1 [file jof-09-00161-s001.zip › jof-2129612-supplementary.pdf]

Diagnosis of Aspergillosis in Horses

Radim Dobiáš [1,2,*,†], Petr Jahn [3†], Katarina Tóthová [3], Olga Dobešová [3], Denisa Višňovská [2,4], Rutuja Patil [5,6], Anton Škríba [5], Pavla Jaworská [1], Miša Škorič [7], Libor Podojil [3], Michaela Kantorová [8], Jakub Mrázek [8], Eva Krejčí [1,2], David A. Stevens[9,10] and Vladimír Havlíček [5,6]

[1] Department of Bacteriology and Mycology, Public Health Institute in Ostrava, 702 00 Ostrava, Czech Republic
[2] Department of Biomedical Sciences, Faculty ofMedicine, University of Ostrava, 703 00 Ostrava, Czech Republic
[3] Equine Clinic, University of Veterinary Sciences, 612 42 Brno, Czech Republic
[4] Department of Biology and Ecology, Faculty of Science, University of Ostrava, 710 00 Ostrava, Czech Republic
[5] Institute of Microbiology of the Czech Academy of Sciences, 142 20 Prague, Czech Republic
[6] Department of Analytical Chemistry, Faculty of Science, Palacký University, 771 46 Olomouc, Czech Republic
[7] Department of Pathology and Parasitology, University of Veterinary Sciences Brno, 612 42 Brno, Czech Republic
[8] Department of Molecular Biology, Public Health Institute in Ostrava, 702 00 Ostrava, Czech Republic
[9] California Institute for Medical Research, San Jose, CA 95128, USA
[10] Division of Infectious Diseases and Geographic Medicine, Stanford University School of Medicine, Stanford, CA 95128, USA
* Correspondence: radim.dobias@seznam.cz

Supplementary Table S1: Review of previously reported cases of invasive pulmonary aspergillosis in horses.

Country	No. of cases in the study	No. of cases/Breed	Age	Sex	Causative agent	Underlying condition	Diagnosis	Outcome	Reference
**Canada**	1	1 SB	20 years	Mare	*Aspergillus fumigatus*	Hepatopathy susp.	PM	death	Long et Mitchel 1971
**USA (N.Y.)**	1	1 SB	2 years	Filly	*Aspergillus* spp.	Myelomonocytic leukemia	PM	euthanasia	Blue et al. 1987
**USA (Florida)**	1	1 QH	7 months	Colt	*Aspergillus fumigatus*	Diarrhea, laminitis, bacterial uveitis	PM	death	Green et al. 1987
**USA (Michigan, N.Y.), Australia**	19	(5 TB,<br>4 QH,<br>2 Arabian,<br>2 Appaloosa,<br>2 Grade,<br>1 Saddlebred,<br>1 Palomino,<br>1 Standardbred,<br>1 Welsh)	1 month – 15 years	(Stallions 8, Mares 8, Geldings 3)	*Aspergillus* spp.<br>(2 of them *Aspergillus fumigatus*)	(Diarrhea – 14,<br>Pneumonia – 3,<br>Umbilical hernia repair – 1,<br>Laminitis – 3,<br>Septicemia – 1,<br>Obstructive urolithiasis – 1,<br>Myelogenous leukemia – 1,<br>Anemia and ataxia – 1,<br>Corticosteroid treatment – 3)	PM	death/ euthanasia	Slocombe et Slauson 1988
**USA (Pennsylvania)**	1	1 QH	8 years	Mare	*Aspergillus* spp.	Enteritis/colitis	PM	death	Hattel et al. 1991
**USA (Ohio)**	1	1 National Show Horse	4 years	Gelding	*Aspergillus* spp.	*Streptococcus zooepidemicus* Infection	IV susp.	euthanasia	Moore et al. 1993
**USA (Missouri)**	1	1 QH	7 years	Gelding	*Aspergillus* spp.	Large colon volvulus (surgery), diarrhea	PM	euthanasia	Pace et al. 1994
**USA (Michigan)**	1	1 TB x QH	11 years	Gelding	*Aspergillus* spp.	Myelomonocytic leukemia	PM	death	Buechner-Maxwell et al. 1994
**Spain**	1	1 French Saddlebred	30 years	Mare	*Aspergillus fumigatus*	Cushing syndrome	PM	euthanasia	Carrasco et al. 1996
**USA (Michigan)**	1	1 QH	6 years	Mare	*Aspergillus* spp.	Enterocolitis	IV susp.	death	Rosenstein et Mullaney 1996
**Spain**	1	1 TB	2 years		*Aspergillus niger*	*Rhizopus solonifer* pneumonia	PM	death	Carrasco et al. 1997
**USA (Pennsylvania)**	29	(10 TB,<br>11 SB,<br>2 QH,<br>2 Western,<br>1 Appaloosa,<br>1 Saddlebred,<br>1 Arabian,<br>1 Pony)	5.0 ± 4.4 years	(Mare 15, Stallion 7, Gelding 7)	*Aspergillus* spp.	[Primary GIT disease – 20,<br>Secondary GIT disease – 5<br>(diarrhea 12, colic 4, weight loss 2, others 1 each: laryngoplasty, renal failure, sinus surgery, pleuropneumonia, myositis, DIC, toxemia, hepatic failure, ataxia, debilitation)]	IV – 1<br>IV susp. – 1<br>PM - 27	euthanasia 17<br>death 12	Sweeney et Habecker 1999
**USA (Missouri)**	2	(1 QH,<br>1 Arabian)	2 - 14 years	(Gelding, Stallion)	*Aspergillus* spp.	[Ataxia (EPM)<br>Strangulation of small intestine (surgery)]	PM<br>PM	death<br>death	Johnson et al. 1999
**Iran**	1	N/A	7 years	N/A	*Aspergillus fumigatus*	Purulent pneumonia	PM	death	Tafti et al. 2001
**France**	1	1 Pony	6 years	Mare	*Aspergillus fumigatus*	Colitis, mucormycosis	PM	death	Thirion–Delalande et al. 2005
**USA (California)**	1	1 TB	14 days	Colt	*Aspergillus fumigatus*	Diarrhoea (enterocolitis)	IV	healing after surgical resection of lesion and voriconazole administration	Hilton et al. 2009
**Brazil**	2	(1 Brasileiro-de-Hipismo,<br>1 PH)	1.5 - 3 years	(Filly 1, Colt 1)	*Aspergillus fumigatus* both horses	Both horses: anaemia, apathy, fever, neurological signs, poor body condition	PM<br>PM	death<br>death	Headley et al. 2014

SB, Standardbred; QH, Quarter Horse; TB, Thoroughbred; PH, Paint Horse; PM, postmortem; IV, *in vitro*; susp., suspicious; EPM, equine protozoal myelitis; N/A, not available

Supplementary Table S2. Calibration curves for gliotoxin and ferricrocin. Calibration linearity and regression coefficients (R^2) are shown in the insets. For quantitation of gliotoxin in bronchoalveolar lavage fluid, the calibration curve (blank, 1, 5, 10, 50, 100, 250 ng/mL) was constructed from [M+H]+ ion signals (**a**). The instrumental LOD and LOQ were 2.1 ng/mL and 6.5 ng/mL respectively. The content of gliotoxin in lung tissue homogenate was determined by use of prepared standards (0, 100, 200, 300, 400 ng/mL) (**b**). Ferricrocin calibration (blank, 1, 5, 10, 50, 100, 250 ng/mL) was plotted using [M+H]+, [M+Na]+, and [M+K]+ ion signals with the instrumental LOD and LOQ, 0.6 ng/mL and 1.9 ng/mL respectively (**c**).

a

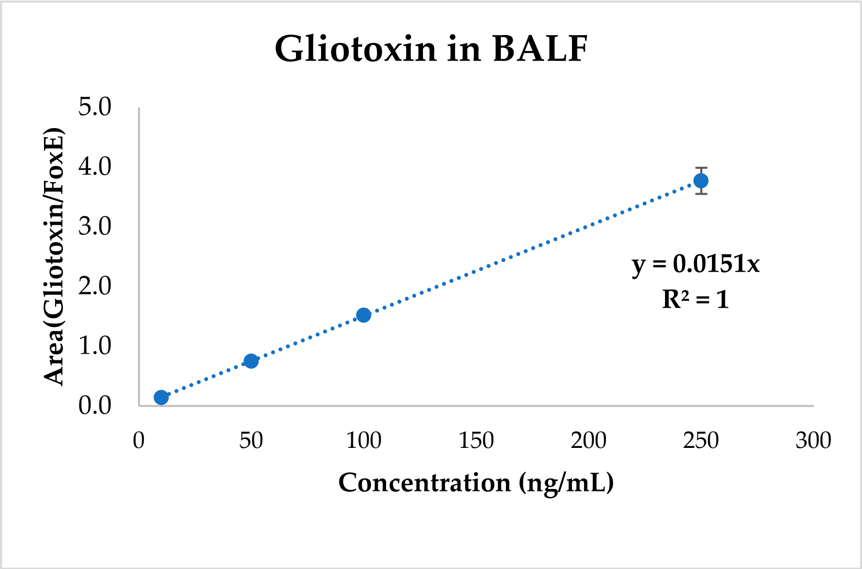

b

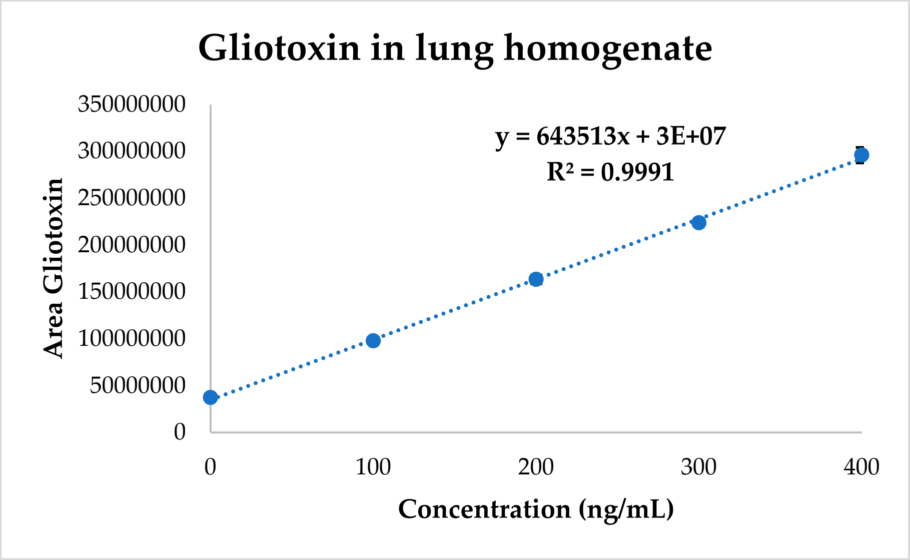

c

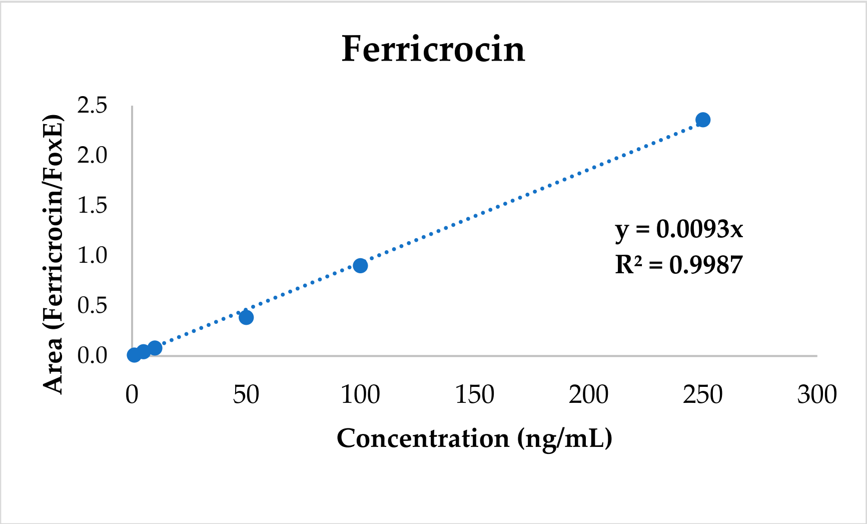

Supplementary Table S3. Characteristics of horses with underlying pulmonary disease, healthy controls and horse with IPA. Results of *Aspergillus* spp. detection in tracheal wash and BALF. IPA, invasive pulmonary aspergillosis; EHV5, Equine Herpes Virus 5; BALF, Bronchoalveolar Lavage Fluid. Difference between the severe asthma, moderate asthma, IPA and the healthy control group with regard to the occurrence of *Aspergillus* spp. in BALF and tracheal wash was statistically significant (P<0.05).

	Total enrolled (n=18)	Severe Asthma (*n*= 8)	Moderate Asthma (*n* = 4)	IPA, EHV 5, Colitis, Death (*n*=1)	Healthy (*n*=5)
Age - mean (range)	12 (3-22)	11.9 (9-20)	11.5 (8-14)	3 (3)	10.2 (3-22)
Stallions (%)	2 (11)	1 (13)	0 (0)	1 (100)	0 (0)
Mares (%)	7 (39)	2 (25)	2 (50)	0 (0)	3 (60)
Geldings (%)	9 (50)	5 (63)	2 (50)	0 (0)	2 (40)
**Tracheal wash culture:**					
Aspergillus fumigatus		4	1	Not sampled	1
Aspergillus montevidensis		2	1	None	None
Aspergillus flavus		1	None	None	None
Aspergillus niger		4	1	None	None
Aspergillus nidulans		1	1	None	None
Aspergillus chevalieri		1	None	None	None
**BALF culture:**					
Aspergillus fumigatus		1	1	1	None
Aspergillus montevidensis		3	1	None	None
Aspergillus flavus		1	None	None	None
Aspergillus niger		1	None	None	None
Aspergillus nidulans		None	None	None	None
Aspergillus chevalieri		1	None	None	None
**Tracheal wash + BALF identical finding:**					
Aspergillus fumigatus		1	1	-	None
Aspergillus montevidensis		2	1	None	None
Aspergillus flavus		None	None	None	None
Aspergillus niger		None	None	None	None
Aspergillus nidulans		None	None	None	None
Aspergillus chevalieri		1	None	None	None
